# Health promotion programs in prison: attendance and role in promoting physical activity and subjective health status

**DOI:** 10.3389/fpubh.2023.1189728

**Published:** 2023-07-21

**Authors:** Riki Tesler, Ofer Regev, Ruth Birk, Sharon Barak, Yair Shapiro, Yossi Weiss, Avi Zigdon, Kathrin Ben Zvi, Yochanan Vaknin, Gizell Green, Idit Sohlberg, Moti Zwilling, Liav Goldstein

**Affiliations:** ^1^Department of Health System Management, School of Health Sciences, Ariel University, Ariel, Israel; ^2^Department of Health System Management, Health Promotion and Wellbeing Research Center, School of Health Sciences, Ariel, Israel; ^3^Department of Nutrition Sciences, School of Health Sciences, Ariel University, Ariel, Israel; ^4^Department of Nursing, School of Health Sciences, Ariel University, Ariel, Israel; ^5^Department of Pediatric Rehabilitation, The Chai Sheba Medical Center, The Edmond and Lily Safra Children’s Hospital, Ramat-Gan, Israel; ^6^Israel Prison Service, Ramla, Israel; ^7^Department of Business Administration, Ariel University, Ariel, Israel; ^8^Israel Prison Service, Medical Officer Office, Ramla, Israel

**Keywords:** prison, health promotion, physical activity, health promotion programs, subjective health status, inmates

## Abstract

**Introduction:**

Maintaining an inmate’s health can serve as a challenge due to unhealthy background, risky behavior, and long imprisonment. This study aimed to analyze the prevalence of participation in health promotion activities among Israeli inmates and its association with their physical activity levels and subjective health status.

**Methods:**

A cross-sectional study was designed to examine 522 inmates (429 males, 93 females). The data were collected by trained face-to-face interviewers and self-report questionnaires.

**Results:**

Most of the participants (82.37%) did not meet the recommended physical activity level. Half of the participants reported that their physical activity levels decreased since they were in prison compared with 29.50% who reported that their physical activity levels increased. Physical activity and subjective health status were significantly higher among younger male inmates. Furthermore, participation in health-promoting activities was associated with higher levels of physical activity and subjective health status.

**Discussion:**

Health promotion activities may play an important role in addressing the challenges of maintaining inmate health. Implications of the findings are further discussed.

## Introduction

The health of inmates is a major concern among health service officials, as this population often suffers from high rates of substance use and exposure to violence. Moreover, inmates tend to come from backgrounds of poverty; low education; and poor professional skills, factors that are consistently associated with reduced physical and mental health (1). More specifically, compared to the general population, inmates suffer from higher rates of contagious diseases such as HIV, hepatitis B and C, and tuberculosis, a higher rate of mental health problems and disorders, and a higher risk of cardiovascular disease and some types of cancer (2). Along with poor incarceration conditions and delays in medical treatment, such factors can lead to the deterioration in both physical and mental health (3–7).

Maintaining the health of people living in prison is not only a matter of equal rights and humanitarian justice, but is of paramount importance to public health; maintaining their health is also a legal requirement, not a luxury (4, 5). Many inmates serve short prison sentences and return to the community where they live (8); therefore, precarious health conditions may carry an additional burden on community health services (2, 3, 6) and the high prevalence of communicable diseases that are not monitored and treated among the inmates can constitute a real hazard to public health (7). Wider benefits of maintaining positive prison health include lowering the costs of incarceration, lowering public health expenditure, improving reintegration into society and reducing recidivism, reducing health inequalities, and reducing prison population size (6, 7). In this sense, the prison framework provides an opportunity for the health system to provide medical care for populations that are difficult to reach within the community (4, 5), while also promoting preventive medicine and encouraging healthy behaviors and lifestyles (3, 7). Prisons have the potential to significantly improve the health, well-being, and life chances of some of society’s most marginalized and excluded members (9).

A review of legislation enacted to advance health and wellbeing in prison environments indicates that much more could be done to improve the health of inmates (6, 8). The prison population has grown in many Western nations in recent years, but the capacity of prison services has not kept up (3, 10). Where health promotion has been developed in prisons, it tends to follow a medical model, focusing on individual lifestyle choices rather than broader determinants of health (6–10).

A socioecological model of health, a salutogenic orientation concerned with what promotes well-being and makes people thrive, a systems perspective, and an emphasis on holistic change are all part of the approach, which recognizes that health is formed and experienced in everyday settings (10, 11). When employed in this situation, the settings approach prioritizes a comprehensive prison perspective, revisits ideas of control, choice, and empowerment, and makes use of a framework that is determinants-focused (9, 10).

The literature shows that health promotion programs can effectively improve inmate health. Health promotion activities such as structured physical activity, nutrition education, and smoking cessation programs were found to be positively correlated with the cardiovascular health of inmates during incarceration (12). Bilderbeck et al. (13) found that inmates who participated in yoga classes reported an increased positive affect and reduced stress and psychological distress levels; the inmates in the experiment group exhibited better cognitive performance than their counterparts in the control group. Other studies have found that yoga and mindfulness meditation improved the well-being and behavioral functioning of the participating inmates (14). Moreover, there is evidence that these health-promoting activities not only improve the well-being and the cognitive functioning of the inmates while incarcerated but also have the potential to reduce recidivism (15), whereas poor mental health during and after incarceration is linked to higher odds of recidivating (16).

In 2020, there were approximately 14,000 inmates located in 33 different prisons throughout Israel. About 30% of the inmates served conviction periods of no more than 2 years, and 40% of them suffered from some chronic medical condition (17). Since 2010, the Israel Prison Service has issued orderly procedures for the health promotion activities to be implemented within the prison population, including physical activity programs (e.g., aerobic exercises, strength training, availability of gyms, and various health education activities) (18).

The current study aimed to assess the prevalence of participation in various health promotion programs, levels of physical activity, and subjective health status among prison inmates in Israel. More specifically, we sought to examine how participation in health promotion programs as well as socio-demographic and imprisonment characteristics of the inmates are related to the inmates’ level of physical activity and subjective health status.

## Methods

A cross-sectional study was conducted from February–September 2019 in 11 prisons in Israel: three prisons in the Northern District, three prisons in the Southern District, and five prisons in the Central District. Data on socio-demographic characteristics, healthy habits, subjective health status, and prison situation variables were collected using a structured questionnaire. Data were collected by trained interviewers (in-person interviews).

### Ethics statement

The studies involving human participants were reviewed and approved by the Institutional Ethics Committee of Ariel University (AU-HEA-RT-30315027). The patients/participants provided their written informed consent to participate in this study.

### Participants and sampling

This study included inmates aged 21 years old and above. Inmates who were critically ill, unable to communicate and security inmates were excluded. A convenience sampling technique was used; 429 male inmates and 93 female inmates participated. Prior to the start of the study, ethical approval was obtained from the institutional review board (IRB) of the university. Informed consent was obtained from the participants by an external researcher who had no affiliation with the research team, ensuring impartiality and minimizing potential biases.

To assist with the data collection, five data research collectors and one supervisor were employed and trained on data collection procedures and ethics in a one-day training session. The English version questionnaires were translated to Hebrew and were then translated back to English to check their translation accuracy (19, 20). The questionnaire was pre-tested on 20 participants (5% of the sample size) in another prison in the region, and modifications were made to the questionnaire based on the evaluation. The internal consistency of the questionnaire was checked using Cronbach’s alpha; the questionnaire had an alpha value of 0.83, showing a good level of internal consistency (α > 0.7).

### Instruments

The questionnaire was comprised of socio-demographic and imprisonment characteristics, a measure of engagement in physical activity, a subjective health status, and an evaluation of participation in health promotion programs according to a self-report on a scale of 1 to 5, where 1 indicated no participation and 5 indicated full participation. Following is a description of the variables.

Socio-demographic questionnaire – Data were collected on participants’ age (18+), sex (male, female), country of origin, level of education, and marital status. Originally, marital status had six response options (single, in a relationship, married, separated, divorced, widowed). However, this variable was dichotomized into “not in a relationship” (single, separated, divorced, widowed) and “in a relationship” (in a relationship and married).

#### Imprisonment characteristics

Participants were asked to report on two imprisonment-related characteristics: years of detention and the number of family visits to the prison during the previous month. In the questionnaire, the latter options were: never, once, 2–4 times, and more than 4. This variable was also dichotomized to indicate “no visit” (never) and “at least one or more visits per month” (once, 2–4 times, more than 4).

#### Participation in health promotion programs

Participants were asked about their tendency to participate in seven health promotion activities: yoga, gym, meditation, Vipassana seminar, Jangling, smoking lectures, and a healthy nutrition seminar. A score of 0 was given to those who did not participate and 1 to those who did participate. Participants were grouped into individual inmates who did vs. did not participate in any of the above health promotion activities. The mean of the health promotion activities was also calculated. Finally, participants were asked whether they thought that there was a need for additional health promotion activities, namely nutrition groups, a variety of physical activity groups, and health lectures.

Physical activity – Participants were asked about their pre-prison and current weekly time spent in moderate to vigorous physical activity (MVPA), including rapid walking, slow walking, running, aerobic training, and gym training, including both aerobic and muscle strengthening activities. Total time spent in MVPA was calculated as the sum of the aforementioned five activities, classified as insufficient MVPA (<150 min/week) and sufficient MVPA (≥150 min/week), according to the physical activity guidelines for health benefits (WHO, 2018). To get greater benefits from MVPA, it is recommended by the World Health Organization to conduct more than 300 min of weekly MVPA (WHO, 2020) Accordingly, the third category of the MVPA was created of those conducting >300 min/week with one or more activities from the above list. To learn about the effect of prison on participants’ physical activity levels, they were asked to report their physical activity levels while in prison compared to their pre-prison levels. Response options were as follows: more active, same, less active, do not know.

Subjective health status (SHS) – SHS was evaluated by asking the following question: “How do you evaluate your health generally?.” The scale included six levels, where 6 = excellent and 1 = very bad. This variable was also dichotomized to create two categories of SHS: “good-to-excellent” and “bad and very bad” health. The scale and the question were adopted from (21).

### Data management and analysis

#### Assessment of normality

An assessment of the normality of the continuous data was conducted using Kolmogorov–Smirnov test. This test is commonly used for n ≥ 50. The test’s null hypothesis stated that data are from a normally distributed population. In the current study, physical activity levels and SHS did not normally distribute (*p* < 0.001). Therefore, non-parametric statistics were used when analyzing these variables.

#### Socio-demographic and imprisonment characteristics

Descriptive statistics (means, standard deviations, ranges, and percentages) for the socio-demographic and imprisonment characteristics were conducted.

#### Physical activity level and subjective health status

Participants’ physical activity levels (minutes of weekly aerobic physical activity) is described using descriptive statistics. Physical activity levels are also presented visually using a boxplot figure. Percentage of participants who reported being more, same, or less physically active since being in prison was calculated as well. The percentage of participants reporting each of the five health status categories (excellent, very good, good, bad, very bad) was calculated and compared using chi-squared tests.

#### Participation and recommendations for health promotion programs

The percentage of inmates who participated, did not participate, or who were recommended to participate in one or more of the given health promotion activities were calculated. In the first step, participants were grouped into those meeting/not meeting physical activity recommendations and those with good-to-excellent health or bad-to-very bad health. Differences between those meeting/ not meeting physical activity recommendations and those with good-to-excellent health or bad-to-very bad health in the continuous variables were examined using the non-parametric Mann–Whitney rank-sum test. Differences between the groups in categorical variables were examined using chi-squared tests. In the next step, variables that statistically significantly differed between the two groups were further analyzed using two separate binary logistic regression models to determine the extent to which demographic characteristics, imprisonment characteristics, and attendance in health promotion programs predicted the physical activity level and SHS. In that respect, the dependent variables (physical activity level and SHS) were recoded as dummy variables (0 = not meeting physical activity recommendations or reporting bad and very bad health, and 1 = meeting physical activity recommendations or reporting good-to-excellent health).

### Power analysis

Post-hoc power analysis was conducted separately for physical activity level and SHS using G*power (version 3.1). For physical activity level, using the Wilcoxon-Mann Whitney test (differences between two independents, namely, those achieving vs. not achieving the recommended physical activity level), two-tails, alpha err probability of 0.05, and with achieved mean effect size d = 0.40, the power achieved in the study was 0.90. For SHS, using the Wilcoxon-Mann Whitney test (differences between two independents, namely, those with good-to-excellent health vs. those with bad-to-very bad health), two-tails, alpha err probability of 0.05, and with achieved mean effect size d = 0.31, the power achieved in the study was 0.88.

The data were analyzed with the IBM SPSS statistics 25. In all statistical analyses, *p*-values lower than 0.05 were considered as statistically significant.

## Results

### Socio-demographic and imprisonment characteristics

Our study included 522 sample of Israeli inmates with a mean age of 38.20 + 12.83 years (males: *n* = 429, 82.2%). The average years of detention was 4.55 + 1.55 years (Table 1).

**Table 1:** Socio-demographic and imprisonment characteristics of the sample (*n* = 522).

Variables			Mean (SD) [range] OR *N* (%)
Socio-demographic characteristics	Age, years: mean (SD) [range]		38.20 (12.83) [18.00–82.00]
	Sex: *n* (%)	Male	429 (82.20)
		Female	93 (17.80)
	Country of origin: *n* (%)	Native to the country	356 (74.01)
		Foreign	125 (25.98)
	Marital status: *n* (%)	Single	173 (33.70)
		In partnership	45 (8.80)
		Married	174 (33.90)
		Separated	19 (3.70)
		Divorced	88 (17.20)
		Widowed	14 (2.70)
	School, years: mean (SD) [range]		9.45 (3.26) [0.00–15.00]
	Educational level: *n* (%)	Never learned at school	28 (6.10)
		Only primary school	86 (18.70)
		Graduating school without diploma	88 (19.10)
		Basic diploma	120 (26.00)
		Professional diploma	56 (12.10)
		College diploma	12 (2.60)
		Technical or professional certification	23 (5.00)
		Professional degree/ course	48 (10.40)
Imprisonment characteristics	Years of detention: mean (SD) [range]		4.55 (1.55) [2.00–15.00]
	Family visitation in the previous month: *n* (%)	Never	158 (30.44)
		Once	161 (31.02)
		2–4 times	140 (26.97)
		More than 4	60 (11.56)

SD, standard deviation.

### Physical activity level

**Figure 1:**
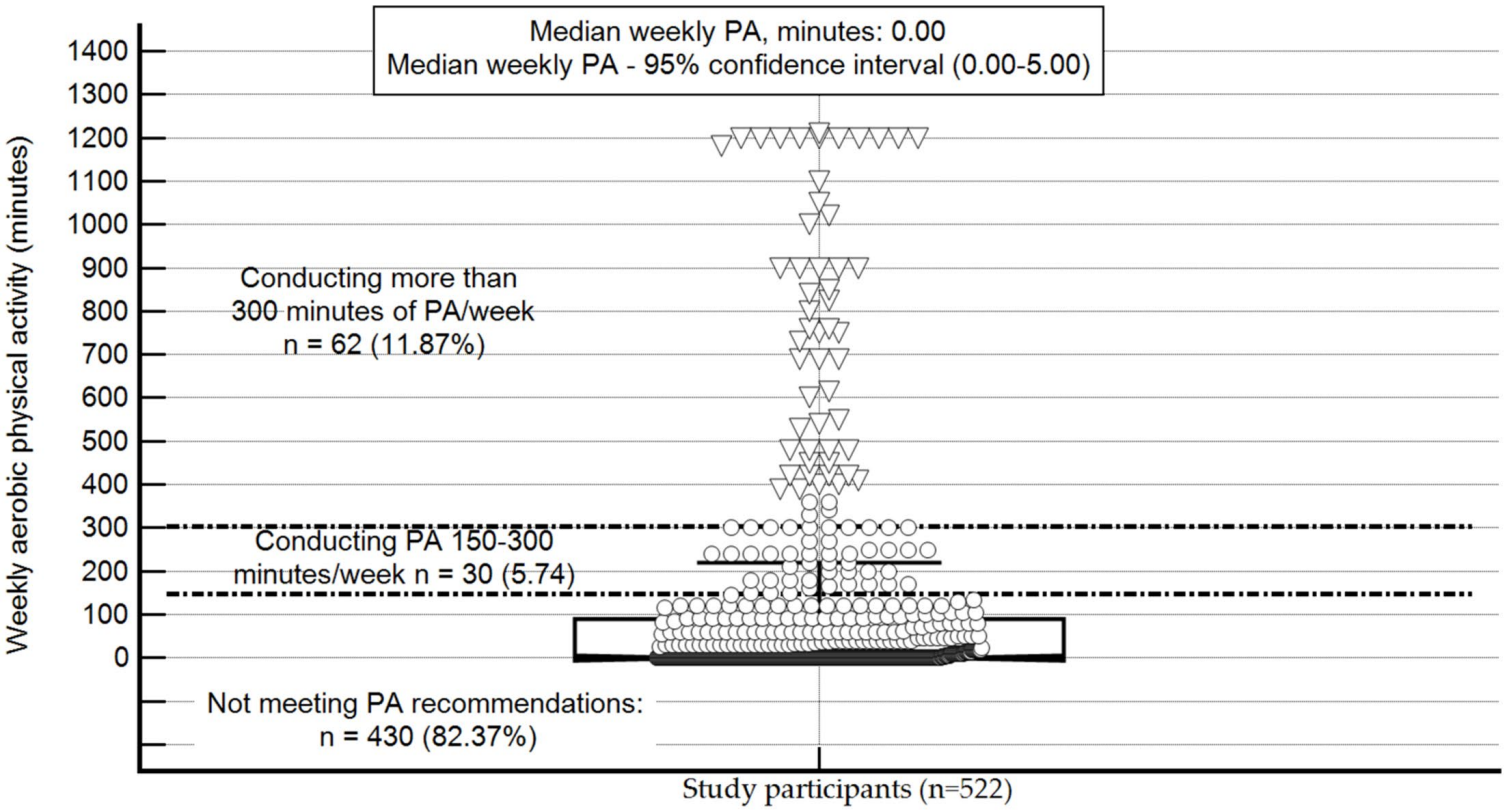
Physical activity level of study participants (*n* = 522), PA, physical activity; The central box represents the values from the lower-to-upper quartile (25-to-75 percentile); the vertical line extends from the minimum-to-the maximum value, excluding outside values (denoted in triangles). Non-outliers are denoted in circles. An outside value is defined as a value that is smaller than the lower quartile minus 1.5 times the interquartile range or larger than the upper quartile plus 1.5 times the interquartile range; the middle line represents the median; The area from the first horizontal line to the *X* axis (weekly aerobic activity of <150 min) represents individuals not meeting the recommended physical activity level. The area between the first and the second horizontal lines (150–300 min of weekly physical activity) represents individuals meeting physical activity recommendations. The area above the third horizontal line (>300 weekly minutes) represents people being sufficiently active to receive extra health benefits from physical activity.

Participants’ MVPA levels varied considerably and ranged from 0 min to 1,210 min (median minutes: 0.00, 95% confidence interval: 0.00–5.00; Figure 1). Most participants did not meet the recommended physical activity level of at least 150 min of weekly MVPA (82.37%). In addition, 29.50% (*n* = 154), 15.51% (*n* = 81), and 49.80% (*n* = 260) of participants reported that their physical activity levels increased, remained the same, or decreased since they entered prison, respectively.

### Subjective health status

The prevalence of excellent, very good, good, bad, and very bad SHS was 17.24 (*n* = 90), 18.58 (*n* = 97), 27.39 (*n* = 143), 23.18 (*n* = 121), and 13.60 percent (*n* = 71; chi-squared = 5.33, *p* = 0.02). Post-hoc test showed that more participants reported good health (*n* = 143, 27.39%) compared to those who reported very bad health (*n* = 71, 13.60%; chi-squared = 25.04, *p* < 0.001).

### Participation in health promotion programs and recommendations for additional health promotion programs

Approximately 43% (*n* = 226) of study participants did not participate in any health promotion program. The most prevalent program was gym (35.63% participation rate) followed by meditation (*n* = 119, 22.79%) and yoga (*n* = 115, 22.03%). Activities with the least enrolled participants were: smoking lectures (*n* = 76, 14.55%) and healthy nutrition seminar (*n* = 71, 13.60%). The mean number of health promotion programs each participant was enrolled in was 1.29 + 1.59. Approximately 80% of the participants recommended adding the following health promotion activities: a variety of physical activity groups (*n* = 413, 79.11%), health lectures (*n* = 411, 78.73%), and nutrition groups (*n* = 407, 77.96%).

### Prediction of physical activity level and SHS

Overall, three variables significantly differed between participants who did vs. did not meet physical activity recommendations and those with good-to-excellent vs. bad-to-very bad health status (Table 2). More specifically, compared to those who met physical activity recommendations, those who did not meet the recommendations were older (Mann–Whitney U = 1771.00, *p* = 0.01) and participants with bad-to-very bad health were older (Mann–Whitney U = 21,229, *p* < 0.001). In addition, compared to females, the prevalence of meeting the physical activity recommendations and the prevalence of good-to-excellent health among males was statistically significantly higher (chi-squared = 13.80, *p* < 0.001; chi-squared = 27.27, *p* < 0.001 respectively). Finally, compared to those not participating in health promotion programs, the prevalence of meeting the physical activity recommendations among those who participated in health promotion activities was statistically significantly higher (chi-squared = 11.39, *p* < 0.001). Accordingly, the prevalence of good-to-excellent health among participants who did not participate in health promotion programs was statistically significantly lower than the level found among those who participated in such programs (chi-squared = 11.30, *p* < 0.001).

**Table 2:** Differences in physical activity level and health status based on demographic, imprisonment characteristics, and participation in health promotion programs.

Variables			Physical activity level		Health status	
			Meeting physical activity recommendations		Self-reported health status	
			Yes: median (95% confidence interval) OR *n* (%)	No: median (95% confidence interval) OR *n* (%)	Good-to-excellent: median (95% confidence interval) OR *n* %)	Bad and very bad: median (95% confidence interval) OR *n* (%)
Socio-demographic characteristics	Age, years: mean (SD) (*n* = 522)		32.00 (30.00–37.00)	37.00* (35.00–38.31)	33.00 (32.00–36.00)	43.00* (39.35–46.00)
	Sex: n (%)	Male (*n* = 429)	88 (20.51)	341 (79.48)	295 (70.07)	126 (29.92)
		Female (*n* = 93)	4 (4.30)^†^	89 (95.69)^†^	35 (41.17)^†^	50 (58.82)^†^
	Country of origin: n (%)	Native to the country (*n* = 356)	67 (18.82)	289 (81.17)	240 (67.41)	116 (32.58)
		Foreign (*n* = 125)	18 (14.4)	107 (85.6)	81 (64.8)	44 (35.2)
	Marital status: n (%)	In a relationship (*n* = 219)	35 (15.98)	184 (84.01)	135 (61.64)	77 (35.15)
		Not in a relationship (*n* = 294)	55 (18.70)	239 (81.29)	194 (65.98)	93 (31.63)
	School, years: mean (SD) (*n* = 461)		10.00 (10.00–11.60)	10.00 (10.00–10.00)	15.00 (10.00–11.00)	15.00 (10.00–10.00)
Imprisonment characteristics	Years of detention: mean (SD) (*n* = 522)		4.500 (4.00–5.00)	4.00 (4.00–4.00)	4.00 (4.00–4.00)	5.00 (4.00–5.00)
	Family visitation in the previous month: *n* (%)	No visitations (*n* = 158)	22 (13.92)	136 (86.07)	93 (58.86)	57 (36.07)
		At least one visitation (*n* = 361)	70 (19.39)	289 (80.05)	237 (65.5)	115 (31.85)
Health promotion programs	Participation status: *n* (%)	Yes (*n* = 296)	66 (22.97)	228 (77.02)	218 (73.64)	78 (26.35)
		No (*n* = 226)	26 (11.50)^‡^	198 (87.61)^‡^	135 (59.73)‡	91 (40.26)^‡^

*Statistical significant differences between the two groups of physical activity level [“Yes” vs. “No” or health status (“Good-to-excellent” vs. “Bad and very bad”)] (*p* < 0.05; 2-tailed) using independent *t*-tests ^†^Statistical significant differences between males and females (*p* < 0.05; 2-tailed) using Chi-squared test; ^‡^Statistical significant differences between those participating and not participating in health promotion programs (“Yes” vs. “No” groups; *p* < 0.05; 2-tailed) using Chi-squared test; SD, standard deviation.

For physical activity level, compared to males, the odds of females being physically active decreased by 77% (95% confidence interval: 1.24, 3.20). Similarly, compared to those not participating in health promotion programs, the odds of those participating in health promotion programs being physically active increased by 99% (95% confidence interval: 1.24, 3.20). For SHS, sex and participation in health promotion programs were also significant predictors with odds ratio equals 0.46 (95% confidence interval: 0.28, 0.75) and 1.66 (95% confidence interval: 1.13, 2.45), respectively. Unlike physical activity level, age also statistically significantly predicted SHS (odds ratio = 0.96, 95% confidence interval: 0.95, 0.97). For additional information, refer to Table 3.

**Table 3:** Summary of multiple binary logistic regression analysis for physical activity level and health status.

Dependent variable	Variables	Coefficient	Odds ratio	Wald	95% confidence interval	*p*
Physical activity level (reference, meeting physical activity recommendations)	Constant	−1.12	–	7.73	–	0.005
	Age, years	−0.01	0.98	3.40	0.96–1.00	0.060
	Sex (reference category: male)	−1.09	0.33	6.11	0.14–0.79	0.010
	Participation in health promotion programs (reference category: not participating)	0.69	1.99	8.22	1.24–3.20	0.004
	*Model summary*		Chi-squared = 23.36, *p* < 0.001, Nagelkerke *R*^2^ = 0.08			
Health status (reference, good-to-excellent health)	Constant	2.06	–	37.11	–	< 0.0001
	Age, years	−0.03	0.96	23.36	0.95–0.97	< 0.0001
	Sex (reference category: male)	−0.77	0.46	9.52	0.28–0.75	0.002
	Participation in health promotion programs (reference category: not participating)	0.51	1.66	6.74	1.13–2.45	0.009
	*Model summary*		Chi-squared = 45.74, *p* < 0.001, Nagelkerke *R*^2^ = 0.12			

## Discussion

The present study assessed the prevalence of participation in various health promotion programs, level of physical activity, and subjective health status among inmates in Israel. We further examined the role of demographic and imprisonment characteristics and participation in health promotion programs in predicting physical activity level and subjective health status. Our findings show that inmate participation in health promotion activities was positively associated with physical activity level and subjective health status. Moreover, although most of the participating inmates (82.37%) did not meet the recommended physical activity level of at least 150 min of weekly MVPA, those who participated in any health promotion activity had a better chance of meeting the recommended physical activity level. Likewise, the odds of reporting good health were 1.5 times higher among the inmates who participated in these programs compared to those who did not participate. These findings provide additional support to the growing body of research suggesting that health promotion programs and activities benefit inmate health and well-being (12–14), as well as the potential to reduce recidivism (15).

It has been generally claimed that the settings approach that the WHO Health in Prisons Program supports presents an opportunity to realize the potential of prisons to embrace health promotion and truly fight health inequities, in spite of the inherent obstacles (8, 9). The fact that various health promotion programs provided in several prisons were found to be positively correlated with the health status of the inmates and their level of physical activity, emphasizes the importance of maintaining the health of the prisoners and minimizing future burden on public health services due to the onset and deterioration of untreated chronic physical and mental health conditions (3, 7).

Age and sex were the demographic variables that were found to significantly predict health status and meeting the physical activity recommendations. Female inmates were less likely to meet the recommended physical activity levels and their reported health status was much poorer compared with male inmates. These findings corroborate previous research which indicates that women in prison have more disease burden (22) and need more health care services than men (6). Moreover, female inmates often come from at risk families and as such, they are more likely to suffer from poor mental health and higher rates of substance abuse compared to male inmates (22, 23). It is possible that the health promotion activities offered currently at the prisons are more focused on the needs of male inmates, which may lead to the deterioration of health problems experienced by female inmates (6). It is important to note, in Israel, as in many countries women report less physical activity as compared to men in the general population (23). Age was also negatively associated with both dependent variables: younger inmates were more likely to meet the recommended physical activity levels and to report better health than older inmates. Although a negative relationship between age and health is expected, compared to older adults living in the communities, older prisoners suffer from early-onset and more rapid progression of (23) many chronic medical conditions and untreated mental illness due to unhealthy lifestyle and inadequate health care (24, 25). These findings further stress the importance of developing and tailoring health promotion programs to the special needs of these two vulnerable groups of female inmates and older inmates (26, 27).

Years of detention, marital status, and family visitation were not found to be important factors in predicting inmate health status and physical activity levels. However, the direction of results may suggest that family visitation is associated with inmate health and activity levels. Indeed, research shows that family visits in prison can benefit the well-being of prisoners and motivate them to maintain good behavior during and after incarceration, however, it depends on the quality of interactions with the visiting family (4, 28, 29).

Finally, despite the positive and encouraging results of the health promotion programs in Israeli prisons, the current study also found that half of the prisoners reported that their level of physical activity had decreased since entering prison compared to 29.5% who reported that they had increased their level of physical activity since prison. Likewise, 43% of the inmates did not take part in any health promotion program. Given that most of the inmates expressed a desire for additional health promotion programs, these findings further stress the need to invest additional efforts and resources to encourage the widest possible participation of all inmates (9, 10, 30).

This study had some limitations that must be considered. The cross-sectional design precludes the inference of causality. While we know that those who participated in the health promotion program also reported higher level of physical activity, these may also be inmates who were previously healthier and more active prior to being in prison. Data were self-reported, which can be subject to bias. Convenience sampling does not represent the perception of all inmates who were in different stages of incarceration or in different stages of rehabilitation. Conducting face-to-face interviews by a trained interviewer can affect the data collection as a result of the relationship between the interviewer and the inmate. However, the sample size was sufficient to gather insights. Recommended future studies should include objective health measures and data, as well as a longitudinal design to evaluate the long-term effect of participation in health promotion programs.

## Conclusion

The study findings emphasize the importance of providing in prison health programs to promote health activities and well-being perceptions to prevent chronic diseases in Israeli inmates. These findings underscore the importance of evaluating health behavior and SHS levels in prison settings. In addition, the significant association between participation in health promotion programs and subjective health status marks the need to explore the underlying factors for health promotion participation in prison. More positive health outcomes for male as opposed to female inmates and for younger as opposed to older inmates highlighted the specific needs of the different inmate population groups.

## Data availability statement

The raw data supporting the conclusions of this article will be made available by the authors, without undue reservation.

## Ethics statement

The studies involving human participants were reviewed and approved by the Institutional Ethics Committee of Ariel University (AU-HEA-RT-30315027). The patients/participants provided their written informed consent to participate in this study.

## Author contributions

RT, AZ, and OR: investigation. RT, RB, YS, YW, and AZ: original draft preparation. SB: formal analysis. KBZ, YV, GG, IS, LG, and MZ: conceptualization, review, and editing. RT, YS, YW: supervision. All authors have read and agreed to the published version of the manuscript.

## Funding

This publication was made possible by grant no. 24376 from Ariel University.

## Conflict of interest

The authors declare that the research was conducted in the absence of any commercial or financial relationships that could be construed as a potential conflict of interest.

## Publisher’s note

All claims expressed in this article are solely those of the authors and do not necessarily represent those of their affiliated organizations, or those of the publisher, the editors and the reviewers. Any product that may be evaluated in this article, or claim that may be made by its manufacturer, is not guaranteed or endorsed by the publisher.
